# Anti-nephrin autoantibodies in post-transplant recurrence of focal segmental glomerulosclerosis: a review

**DOI:** 10.1080/0886022X.2026.2707051

**Published:** 2026-08-05

**Authors:** Yuhong Ye, Fei Liu

**Affiliations:** Department of Nephrology, Children’s Hospital, Zhejiang University School of Medicine, National Clinical Research Center for Children and Adolescents’ Health and Diseases, Hangzhou, Zhejiang Province, China

**Keywords:** Nephrin, autoantibody, transplantation, recurrence, focal segmental glomerulosclerosis (FSGS)

## Abstract

Emerging studies have identified autoantibodies targeting the podocyte protein nephrin in patients with post-transplant recurrent focal segmental glomerulosclerosis (FSGS). These antibodies bind nephrin, directly disrupting its downstream signaling pathways. This disruption profoundly impacts podocyte structure and function, thereby enriching our understanding of antibody-mediated podocytopathies and their classification. The presence of these autoantibodies correlates with disease activity and offers considerable potential as an innovative biomarker for post-transplant recurrent FSGS. However, the precise contribution of these autoantibodies to the development of recurrent FSGS after transplantation remains elusive. In this review, we summarize the current research on anti-nephrin autoantibodies and discuss their applicability as a new biomarker for diagnosing, predicting outcomes, and guiding treatment in post-transplant recurrent FSGS.

## Introduction

Recurrence of focal segmental glomerulosclerosis (FSGS) is common after kidney transplantation, with reported incidence rates ranging from 6% to 57% [1]. A recent multinational initiative, the Post-Transplant Glomerular Disease (TANGO) study, indicated a recurrence rate of 32% in a multiethnic cohort [2]. Identified risk factors include: a younger age at FSGS diagnosis in the native kidney, a faster progression from FSGS diagnosis to end-stage renal disease (ESRD) <3 years), higher proteinuria at transplantation, an FSGS recurrence in a previous transplant, Caucasian race, and steroid-resistant nephrotic syndrome [1,3,4]. Notably, the most reliable predictor is a history of FSGS recurrence in a previous allograft [1]. Post-transplant recurrent FSGS is associated with poor allograft survival, and the therapeutic approaches remain largely experience-based. Although the mechanisms underlying primary FSGS remains incompletely understood, both experimental and clinical evidence historically implicated circulating permeability factors in the pathogenesis of recurrent FSGS, such as soluble urokinase-type plasminogen activator receptor (suPAR), cardiotrophin-like cytokine factor-1 (CLCF-1), CD40 axis, and apolipoprotein A-Ib (ApoA-Ib) [5]. More recently, anti-podocyte antibodies (APAs) targeting antigens like UCHL1, annexin A2, PSMA1, vinculin, podocin, and Kirrel1 have been identified in minimal change disease (MCD) and FSGS [6–10]. Among these, anti-nephrin Abs have emerged as the most promising pathogenic candidate, with pathogenicity confirmed through clinical investigations, animal models, and *in vitro* studies [11–14].

### The role of anti-nephrin autoantibodies

Watts et al. reported a 27-year-old patient with childhood-onset, steroid-dependent MCD, absence of genetic abnormalities, progressed to ESRD and subsequently underwent kidney transplantation [11]. This patient developed massive proteinuria shortly after transplantation, coinciding with high pre-transplant levels of anti-nephrin Abs. Administration of plasmapheresis and rituximab led to prolonged remission. Circulating anti-nephrin Abs were eliminated and stayed undetectable during 1 year of follow-up. This initial case first revealed the possible relationship between anti-nephrin Abs and post-transplant recurrent podocytopathy.

Consistent with these results, Hattori et al. identified anti-nephrin Abs in the plasma of a patient with post-transplant recurrent FSGS [15]. Furthermore, immunofluorescence analysis of a 1-hour post-transplant biopsy from this patient revealed punctate IgG deposits that specifically colocalized with nephrin. To validate these preliminary findings from the above single patient, this research team subsequently conducted a cohort study [16]. In this multi-institutional study, the elevated plasma anti-nephrin antibody levels were detected in 11 patients experiencing post-transplant recurrent FSGS, measured either before transplantation or during disease recurrence. Graft biopsies during recurrence revealed punctate IgG deposits co-localizing with nephrin. After clinical remission, plasma anti-nephrin antibody levels decreased. Correspondingly, post-remission graft biopsies showed an absence of IgG signals and a restoration of the normal nephrin expression pattern. Together, these findings suggested that circulating anti-nephrin Abs represent a potential pathogenic factor involved in post-transplant recurrent FSGS.

A separate team recently described a kidney transplant patient with recurrent nephrotic syndrome due to podocytopathy who tested positive for anti-nephrin Abs [17]. Complete remission was induced by combination therapy comprising low-density lipoprotein apheresis (LDL-A), steroid pulses, and a single low dose of rituximab. Moreover, anti-nephrin antibody titers correlated with proteinuria severity, confirming the significant pathogenic role for the antibody in this patient’s podocytopathy.

Given the pathogenic implications of anti-nephrin antibodies, researchers have also explored their ability to forecast post-transplant FSGS recurrence. A retrospective multi-center study was carried out to determine whether pre-transplant circulating Abs could predict recurrent FSGS in in a well-defined cohort of kidney transplant recipients [18]. Positive anti-nephrin Abs were identified in 8 of 21 (38%) patients with recurrent FSGS/diffuse podocytopathy, compared to 0 of 17 patients without recurrence (*p* = 0.005). Additionally, patients with positive pre-transplant anti-nephrin Abs (*n* = 8) demonstrated a trend toward worse allograft survival compared to Abs-negative patients. Immunofluorescence analysis revealed punctate IgG deposits co-localizing with nephrin in 5 of 16 (31%) recurrent patients biopsied, whereas biopsies from non-recurrent patients mainly showed the absent IgG staining with preserved continuous nephrin expression. This investigation reinforced the association between anti-nephrin Abs and recurrent FSGS.

Habbig et al. reported on the monitoring and successful pre-transplant removal of anti-nephrin antibodies in an adolescent with primary FSGS scheduled for living-donor kidney transplantation [19]. They documented this young patient with primary FSGS and detectable anti-nephrin antibodies before transplantation. Through preemptive treatment with rituximab and plasma exchange, antibody levels were successfully reduced before transplantation. After transplantation, the patient sustained excellent allograft function with no evidence of disease recurrence over a follow-up of more than 1 year. This case highlights the possibility of anti-slit diaphragm antibody monitoring and pre-transplant elimination as a personalized management strategy for primary FSGS patients requiring kidney transplantation.

### Act alone or in synergy?

Besides anti-nephrin Abs, other podocyte antibodies have also been reported in MCD and FSGS [20]. Similar to systemic lupus erythematosus or membranous nephropathy, anti-nephrin antibodies and other podocyte antibodies might act synergistically in the disease pathogenesis, possibly through a mechanism known as epitope spreading [21]. SLE is always accompanied by the production of several Abs, Arbuckle et al. observed that patients identified an average of 1.5 out of 7 SLE-related autoantigens before diagnosis, increasing to 3 of 7 autoantigens at clinical diagnosis, demonstrating progressive epitope spreading [22]. These findings suggest intermolecular B-cell epitope spreading occurs during the transition from preclinical to clinical autoimmune manifestations, contributing to SLE pathogenesis. In membranous nephropathy, occasional reports indicate dual antigen positivity, such as PLA2R and THSD7A, PLA2R with EXT1/2, THSD7A with EXT1/2, and NCAM1 with EXT1/2. Moreover, clinical cases of membranous nephropathy associated with autoimmune thyroiditis and Graves’ disease illustrate the intermolecular spreading, where Abs against both thyroid and kidney antigens suggests that autoimmunity originating in one organ can lead to the development of autoimmunity in another through epitope spreading [23].

### Standardization and optimization of detection methods

Shirai et al. optimized ELISA methods to detect circulating anti-nephrin Abs while minimizing false positives [24]. They compared three antigen preparations in recurrent FSGS patients and controls: Mouse-derived (Murine non-secreting cell line, NSO cell) recombinant nephrin extracellular domain (ECD), the NSO antigen preparation following pre-adsorption with porcine all-cell pellet (ACP) to remove anti-galactose-α-1,3-galactose antibodies, and Human-derived (HEK293 cell) recombinant nephrin ECD. Of note, the ACP procedure used by Shirai et al. is a serum pre-adsorption step to remove anti-galactose-α-1,3-galactosidase antibodies, not a modification of the antigen itself. Their findings indicated that pre-adsorption with porcine ACP reduced nonspecific IgG binding in the NSO-based ELISA. Furthermore, the HEK293 antigen yielded even lower background signals. Using the HEK293 antigen, 91% (10/11) of rFSGS patients tested positive—a rate markedly higher than the 38% positivity previously reported by Batal et al.^[18]^ These results indicate that HEK293-derived nephrin ECD is superior for ELISA-based detection of anti-nephrin antibodies, effectively minimizing interference from species-specific glycosylation (e.g., galactose-α-1,3-galactose) (Table 1). It is important to recognize that anti-nephrin antibody testing is still undergoing standardization; multi-center validated cutoffs, ROC analyses, inter-laboratory reproducibility, and positive/negative predictive values have not yet been firmly established. Therefore, the performance characteristics reported in Table 1 should be interpreted with caution.

**Table 1.** Comparison of anti-nephrin antibody detection methods.

Method	Authors	Antigen Source	Tag	Sample type	Pretreatment	Cutoff setting	Confirmatory method	Sensitivity	Specificity	Advantages	Limitations
Conventional ELISA	Watts et al. [11] Shirai et al. [16]	NSO (mouse)	His-tag (common)	Serum	None	Arbitrary (OD ratio)	None	Low	Low	Low cost, high throughput	High background, false positives
ELISA with ACP	Watts et al. [11] Shirai et al. [24]	NSO (mouse)	His-tag	Serum	Serum pre-adsorption with porcine ACP	Arbitrary (OD ratio)	None	Moderate	Moderate	Reduced nonspecific binding	Complex preparation
HEK293-based ELISA	Watts et al. [11] Hengel et al. [12] Shirai et al. [24]	HEK293 (human)	Tag-free or minimal	Serum	None	Cohort-derived (ROC not yet validated)	None	Reported high sensitivity in selected cohorts	Reported high specificity in selected cohorts	Low background, high specificity	Higher cost
Immunoprecipitation-Western Blot (IP-WB)	Watts et al. [11] Hengel et al. [12,14]	HEK293 (human)	Tag-free	Plasma	None	Visual detection of specific band	None	Reported high sensitivity in selected cohorts	Reported high specificity in selected cohorts	Potential reference method (requires further validation), low artifacts	Labor-intensive, low throughput
Magnetic bead IP-ELISA	Watts et al. [11] Hengel et al. [12]	HEK293 (human)	Tag-free or biotin	Plasma	None	Quantitative (MFI)	None	Reported high sensitivity in selected cohorts	Reported high specificity in selected cohorts	Good balance of sensitivity/speed	Requires specialized equipment
Immunofluorescence (tissue)	Watts et al. [11]	Human kidney tissue (native or allograft)	N/A	Human kidney tissue	IgG purification to remove plasma inhibitors	Visual colocalization with nephrin	Tissue IgG-nephrin colocalization	Moderate	Reported high specificity in selected cohorts	Visual confirmation, colocalization	Requires IgG purification

Liu et al. systematically evaluated technical challenges and standardization approaches for anti-nephrin autoantibody detection [25]. They compared five methodologies: conventional ELISA, immunoprecipitation followed by Western blotting (IP-WB), magnetic bead-based IP-ELISA, and cell/tissue immunofluorescence. Seven nephrin antigen variants were tested (full-length/truncated forms, His-tagged/FLAG-tagged, biotinylated, mouse-derived (NSO cells)/human-derived (HEK293 cells)) across 169 samples (including FSGS, MCD, SLE, and controls). Their findings revealed: 1. mouse-derived antigens (NSO cells) resulted in high false-positive rates, even in healthy controls. 2. human-derived antigens (HEK293 cells) demonstrated superior reliability. 3. His-tagged antigens induced significant false positives (e.g., T1D samples bound the His-tag rather than nephrin). 4. IP-WB was the most reliable method, identifying two strong-positive FSGS cases and showed weak positivity in about 50% of MCD samples. 5. Conventional ELISA suffered from high background noise. 6. Immunofluorescence required IgG purification before testing, as plasma proteins inhibited antibody binding.

The key consensus of these two studies demonstrated that HEK293-derived antigens are considerably better than mouse-derived antigens due to reduced nonspecific binding, confirming the pathological significance of anti-nephrin Abs in FSGS/MCD. However, discrepancies exist: Shirai et al. reported 91% positivity in recurrent FSGS using HEK293-based ELISA, whereas Liu et al. observed a lower positivity rate. Importantly, the 91% figure from Shirai et al. was obtained with human-derived antigen and is not due to mouse-antigen false positives. The discrepancy likely reflects differences in study populations—Shirai’s cohort predominantly comprised pediatric and Asian patients, whereas Liu’s cohort included more adults and diverse ethnicities. It is possible that anti-nephrin antibody positivity rates are higher in pediatric patients and Asian populations, an issue that warrants further investigation.

Methodologically, while Shirai endorsed HEK-based ELISA as the best option, Liu advocated for IP-WB or magnetic bead IP-ELISA as superior approaches. Notably, Liu’s study revealed critical tag-related artifacts (e.g. His-tag-induced false positives) not addressed by Shirai, providing synergistic insights for future assay optimization. Collectively, both studies contribute significantly to standardizing anti-nephrin Abs detection through refined antigen source selection, methodological refinements, and strategies to reduce the artifacts.

### Current treatment approaches for anti-nephrin-associated recurrence

Treatment options for post-transplant FSGS include preventive measures before transplantation or recurrence, as well as treatment after recurrence. Currently, no universally effective specific therapy exists, and available treatments show variable effectiveness among patients. These include plasmapheresis and rituximab, used either preemptively or after recurrence. Another considered regimen combines plasmapheresis with cyclophosphamide. Various immunosuppressive strategies have also been attempted, such as cyclosporine, abatacept, belatacept, and ACTHar gel [5,6].

A recent cohort study assessed prophylactic treatment in high-risk patients—defined by prior recurrence of FSGS in a previous graft, which supports the primary nature of the disease [26]. This study concluded that prophylactic treatment should not be routinely used in patients undergoing a second transplant after a prior recurrence. No significant differences were observed in overall recurrence rates or 5-year graft survival between those who received prophylaxis and those who did not. In contrast, another study indicated that perioperative therapeutic plasma exchange and rituximab may prevent early recurrence of FSGS and subsequent graft loss, without increasing infection risks [27].

Considering the potential function of Abs in FSGS, therapies targeting antibodies are being explored. Rituximab has been associated with reduced anti-nephrin antibody levels and clinical remission in the selected cases of minimal change nephrotic syndrome and FSGS [12,28]. Case report and case series suggest that ofatumumab may be a safe and effective option for post-transplant recurrence of FSGS [29–34].

Recent reports have shown that daratumumab, a CD38-targeting monoclonal antibody, can deplete antibody-secreting plasma cells and reduce anti‑nephrin antibody titers, leading to remission in some patients with recurrent FSGS [35–36].

It is important to note that the efficacy of rituximab or ofatumumab in post-transplant recurrent FSGS may result from multiple mechanisms, including B-cell depletion, direct podocyte stabilization, and broader immunomodulation; therefore, these responses should not be overinterpreted as specific to anti-nephrin antibody positivity. Moreover, recent evidence indicates that patients with post-transplant recurrent FSGS may harbor autoantibodies not only against nephrin but also against podocin and Kirrel1 [37]. Thus, the disease may be driven by a combination of autoantibodies rather than a single specificity, and future therapeutic strategies may need to target multiple antigens.

Treatment with abatacept or belatacept (B7-1 antagonists) also appears promising, though evidence remains controversial [38–43]. Abatacept and belatacept block the B7-1/CD28 co-stimulatory pathway, which is critical for T-cell activation and subsequent autoantibody production. Case reports have shown variable efficacy: Some patients achieved remission with abatacept [38,41], while others did not respond [40,43]. The mechanism may involve reducing T cell-mediated B cell activation, thereby decreasing anti-nephrin antibody secretion.

### Future perspectives: cellular therapies

Chimeric Antigen Receptor (CAR)-T cell therapy has been employed in lupus nephritis, kidney transplant patients with post-transplant lymphoproliferative disorder (PTLD), and sensitized kidney transplant patients [44–46], whereas no trials have been made in post-transplant recurrence of FSGS. Lately, novel strategies have expanded the potential of CAR-based therapies for autoimmune diseases. Among these, chimeric autoantibody receptor (CAAR) T cells are designed to selectively remove the autoantigen-specific B cells, which improve treatment specificity for autoantibody-driven autoimmune disorders. This strategy has the potential to reduce off-target effects and preserve nonpathogenic B cells that are vital for normal immune function [47,48]. For example, in pemphigus vulgaris (PV)—caused by anti-desmoglein 3 (Dsg3) autoantibodies, mouse studies demonstrated that CAAR T cells engineered with Dsg3 linked to CD137–CD3ζ signaling domains can expand, persist, and effectively deplete Dsg3-specific B cells *in vivo* [47]. A clinical trial of Dsg3-CAAR T cells in patients with mucosal-dominant pemphigus vulgaris is currently ongoing (NCT04422912). Similar CAAR T-cell approaches are being developed for other autoimmune conditions, including myasthenia gravis (MuSK-CAAR T cells) [49], immune thrombocytopenia (GPIbα-CAAR T cells) [50], autoimmune encephalitis (NMDAR-CAAR T cells) [51], lupus nephritis (DNA4-CAAR T cells) [52], and multiple sclerosis (MBP-CAAR T cells) [53].

Beyond CAR T cells, CAAR natural killer (NK) cell therapy has also been investigated in kidney diseases, like membranous nephropathy. One team developed CAAR-NK92 cells and primary human CAAR T cells expressing major membranous nephropathy antigens (PLA2R1 and THSD7A). These cells specifically eliminated PLA2R1- and THSD7A-autoantibody-secreting cells *in vitro*, supporting the feasibility of this approach [54].

Based on the above studies, CAR T or CAAR NK cell therapy targeting the clearance of anti-nephrin Abs may be effective for MCD or FSGS patients positive for these antibodies. Although no related clinical reports have been published to date, these strategies warrant further investigation in cases of post-transplant recurrent FSGS with anti-nephrin antibody positivity.

### Clinical applicability and practical recommendations

When establishing a diagnosis of primary FSGS in a patient being considered for transplantation, it is essential to incorporate: (1) electron microscopic evidence of diffuse foot process effacement, (2) tissue IgG-nephrin colocalization on native kidney or allograft biopsy (if available), and (3) genetic testing to exclude hereditary FSGS. These elements help confirm the autoimmune nature of the disease and guide anti-nephrin antibody testing. The growing evidence positions anti-nephrin antibodies not only as a pathogenic mediator but also as a promising biomarker for recurrent FSGS. Translating this research into clinical practice requires a structured approach to testing, interpretation, and therapeutic decision-making.

#### Who to test and when?

Routine screening for all waitlisted patients with FSGS is not yet universally suggested; nonetheless, testing could be strongly considered in high-risk patients, defined by:

- A history of recurrent FSGS in a previous transplant.
- Rapid progression from native kidney FSGS diagnosis to ESRD (<3 years).
- Pediatric-onset disease.
- Significant pre-transplant proteinuria.

Testing could be performed pre-transplant to stratify the recurrence risk and guide preemptive strategies. Post-transplant, serial monitoring is recommended in high-risk patients or in any patient presenting with new-onset proteinuria, to aid in diagnosing recurrence and differentiating it from other causes of allograft dysfunction.

#### How to test: methodological considerations

As detailed in Section 2.2, the choice of detection method is critical to avoid false-positive results. HEK293-cell-derived recombinant nephrin is superior to mouse-derived antigens due to reduced nonspecific binding from anti-galactose-α-1,3-galactose antibodies. The most reliable methods currently include immunoprecipitation followed by Western blot (IP-WB) and HEK293-based ELISA. Clinicians should inquire about the antigen source and methodology used by their laboratory when interpreting results.

#### Interpreting results and guiding therapy

**Figure 1.**
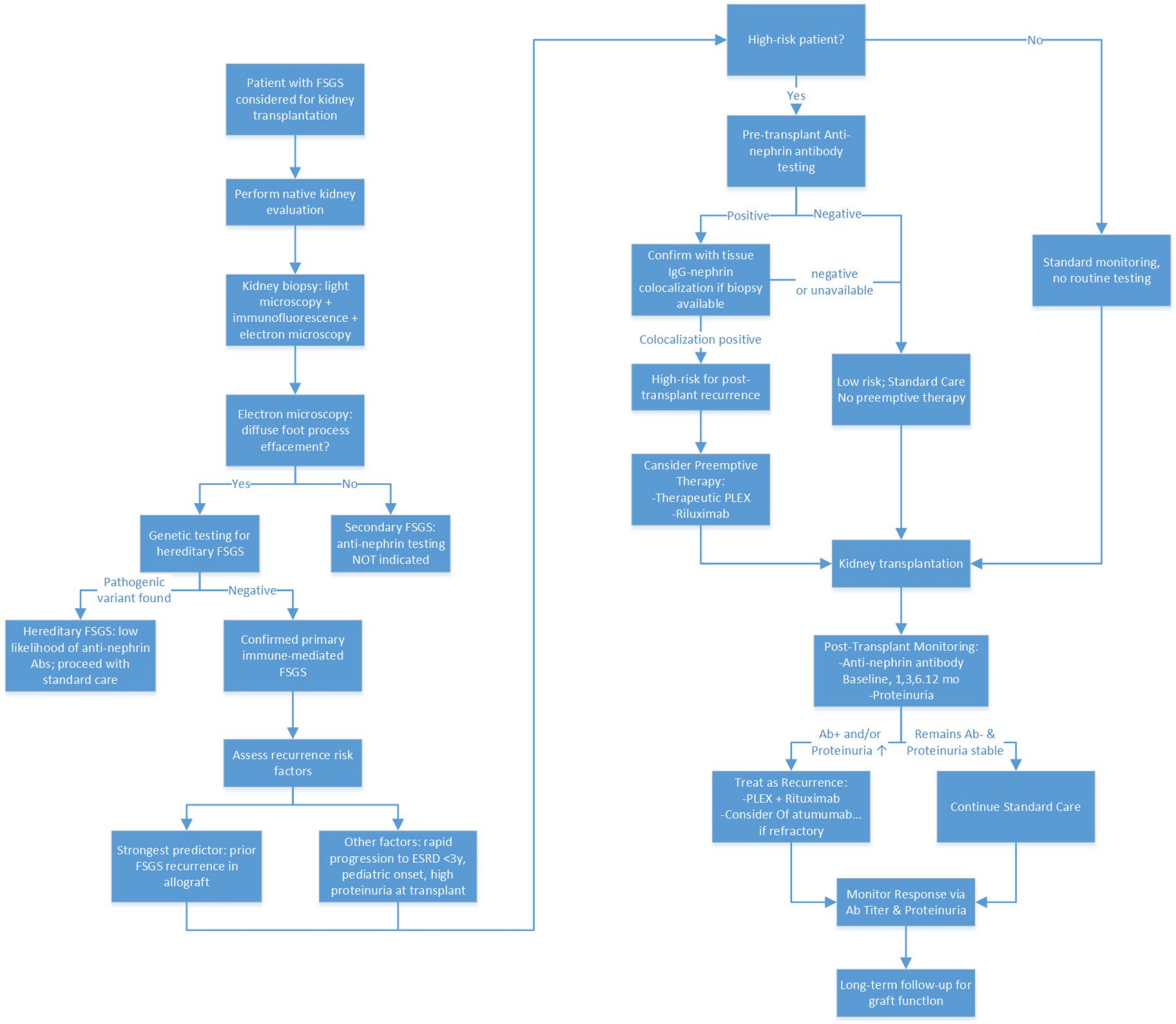
Proposed clinical management algorithm for high-risk FSGS patients based on anti-nephrin antibody status.

A positive anti-nephrin antibody test, especially with a high titer, is associated with a significantly increased risk of post-transplant recurrence. This result can inform a personalized management strategy, as illustrated in the algorithm below (Figure 1).

#### Correlation with treatment response

Anti-nephrin antibody levels exhibit a dynamic correlation with treatment response. A decline in titers following interventions like plasmapheresis or B-cell depletion therapy (e.g. rituximab, ofatumumab) often precedes or accompanies clinical remission. Conversely, persistent or rising antibody levels may indicate refractory disease and the need for intensified or alternative treatment strategies. Thus, serial monitoring serves as a valuable tool for gauging therapeutic efficacy.

## Conclusion

The role of Abs in immune-related diseases has been widely investigated, and the clinical significance and mechanisms of anti-nephrin Abs in MCD and FSGS have been preliminarily explored. A clinical trial has been conducted to evaluate the levels of circulating anti-nephrin autoantibodies in patients with INS, including those with MCD/FSGS and individuals with post-transplant relapse of FSGS (NCT06334692). Although uncertainties remain, anti-nephrin Abs show potential value in supporting diagnosis, managing, and predicting outcomes of post-transplant recurrent FSGS. Future research should focus on standardizing antibody detection methods and validating the clinical significance of these Abs in large cohorts.

## Data Availability

This study did not generate any new datasets. All data analyzed are from publicly available sources, as cited in the manuscript.
